# Turning Characteristics of the More-Affected Side in Parkinson’s Disease Patients with Freezing of Gait

**DOI:** 10.3390/s20113098

**Published:** 2020-05-30

**Authors:** Hwayoung Park, Changhong Youm, Myeounggon Lee, Byungjoo Noh, Sang-Myung Cheon

**Affiliations:** 1 Biomechanics Laboratory, College of Health Sciences, Dong-A University, Busan 49315, Korea; app00113@dau.ac.kr (H.P.); ssam011@dau.ac.kr (M.L.); 2 Department of Healthcare and Science, College of Health Sciences, Dong-A University, Busan 49315, Korea; bnoh@dau.ac.kr; 3 Department of Neurology, School of Medicine, Dong-A University, Dongdaesin-dong 3-ga, Seo-gu, Busan 49315, Korea; smcheon@dau.ac.kr

**Keywords:** Parkinson’s disease, freezing of gait, gait, turning, fall, kinematics

## Abstract

This study investigated the turning characteristics of the more-affected limbs in Parkinson’s disease (PD) patients in comparison with that of a control group, and in PD patients with freezing of gait (FOG; freezers) in comparison with those without FOG (non-freezers) for 360° and 540° turning tasks at the maximum speed. A total of 12 freezers, 12 non-freezers, and 12 controls participated in this study. The PD patients showed significantly longer total durations, shorter inner and outer step lengths, and greater anterior–posterior (AP) root mean square (RMS) center of mass (COM) distances compared to those for the controls. The freezers showed significantly greater AP and medial-lateral (ML) RMS COM distances compared to those of non-freezers. The turning task toward the inner step of the more-affected side (IMA) in PD patients showed significantly greater step width, total steps, and AP and ML RMS COM distances than that toward the outer step of the more-affected side (OMA). The corresponding results for freezers revealed significantly higher total steps and shorter inner step length during the 540° turn toward the IMA than that toward the OMA. Therefore, PD patients and freezers exhibited greater turning difficulty in performing challenging turning tasks such as turning with an increased angle and speed and toward the more-affected side.

## 1. Introduction

Parkinson’s disease (PD) is one of the most common neurodegenerative disorders [1]. A total of 60%–80% of patients with PD eventually develop the freezing of gait (FOG) [1], which is defined as the “episodic absence or marked reduction in forward progression of the feet despite the intention to walk” [2]. The FOG is most commonly experienced during turning tasks, gait initiation, walking through narrow passages, timed up and go (TUG) tasks, dual tasks, and while approaching a destination or avoiding an obstacle [3]. In particular, PD patients with FOG (freezers) encounter greater challenges and need to pay greater attention when performing turning tasks [4]. They face increased risk of falls caused by the instability of their body because it requires the center of mass (COM) to momentarily shift outside the lateral boundaries of the base of support [5].

Previous studies have reported the turning characteristics of 0°, 90°, 120°, and 180° turns [6]; 180° and 360° turns while walking [7]; and 180° turns while walking and 360° turns in place with a narrower range [1] in PD patients and freezers. These studies have revealed that freezers encounter greater difficulty in performing turning tasks than non-freezers, as evidenced by the higher number of steps, slower turning speed, shorter step length, and wider step width with an increasing turning angle. In addition, freezers impose greater demand on lower limb coordination and experience an increased risk of falling and frequent freezing episodes in daily life [6,7].

More than 50% of PD patients exhibit motor impairments, in which unilateral predominance may persist throughout the progression of the disease [8,9]. However, most patients present bilateral impairments at a later stage of the disease [10]. One reason for the unilateral predominance of disease symptoms appears to be the differences in the striatal uptake between the caudate and putamen nuclei [11]. Additionally, the dominant cerebral hemisphere extensively distributed throughout the circuitry of basal ganglia [12]. The asymmetrical gait characteristics of PD patients affect both the upper and lower limbs, and they appear to affect balance control during turning tasks [13]. However, although PD patients show unilateral predominance of disease symptoms, turning tasks assigned in studies so far have been used to evaluate the preferred turning direction toward one side or both sides, without considering the turning task in the direction of the more-affected side.

Spildooren et al. [14] reported that freezers showed higher cadence while turning toward the more-affected side compared to non-freezers. Their findings suggested that freezers experienced greater turning difficulty when turning toward the more-affected side. However, it is still unclear whether there is a difference in the turning characteristics while turning toward the more-affected side in PD patients and freezers. Although PD patients and freezers are affected by the turning speed [15,16], there has been limited research on the characteristics of turning tasks performed at the maximum speed.

Several studies have shown that greater attention and postural stability are required when increasing the turning angle [1,16]. An experimental choice was made to select a 540° turning angle as the challenging condition that would threaten posture stability. This study investigated the characteristics of the turning direction toward the more-affected side in PD patients in comparison with controls, and in freezers in comparison with non-freezers for 360° and 540° turning tasks at the maximum speed. We hypothesized that, for the turning direction of the more-affected side, the turning characteristics with regard to the turning direction may significantly differ between PD patients and controls, and freezers and non-freezers.

## 2. Materials and Methods

### 2.1. Participants

**Figure 1:**
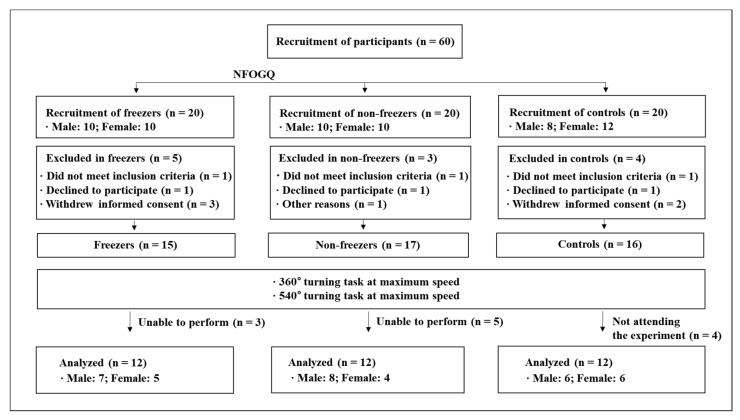
Consort flow diagram. NFOGQ: New freezing of gait questionnaire.

A total of 60 PD patients were recruited. However, 36 of them could not be considered because of reasons such as the withdrawal of informed consent, refusal to participate in the experiment, or inability to participate in the experiment. Eventually, 24 PD patients, which included 12 freezers and 12 non-freezers, were considered, and 12 age-matched older adults also participated as controls. The consort diagram containing the details of the study participants is shown in Figure 1 and Table 1. Idiopathic PD patients were diagnosed by a neurologist using the UK Parkinson’s Disease Society Brain Bank criteria [17]. The inclusion criteria were: (a) Age 50–75 years; (b) capable of walking and moving independently and with a modified Hoehn and Yahr (H&Y) stage of 2–3 [18,19]; (c) mini-mental state examination (MMSE) score of >24 [20]; (d) stable response to antiparkinsonian medications; and (e) classified as freezers with a score of >3 according to the new freezing of gait questionnaire (NFOGQ) [21].

None of the participants (PD patients and controls) reported any history of musculoskeletal injuries or other cardiovascular and neurological diseases in the past six months. The PD patients that required movement assistive devices and those with dyskinesia (uncontrollable muscle movements induced by drug therapy) were excluded. All participants provided written informed consent before participating in this study. The experimental protocols were approved by the institutional review board (IRB) of Dong-A University Medical Center (IRB number: DAUHIRB-17-033 (See Supplementary file S1. IRB number)).

### 2.2. Test Procedures

The PD patients were in the “On” state, which was induced by dopaminergic medication that was administered approximately 2–3 h before the tests. They were sufficiently under the effect of the medication [22]. The experiments were performed in two sessions. In the first session, the participants completed the informed consent form and were assessed according to the Unified Parkinson disease rating scale (UPDRS) [23], modified H&Y stage, NFOGQ, and MMSE (Table 1). In the second session, the participants warmed up for approximately 5 min before performing the turning tasks and practiced such tasks. After 5 min of rest, the test started, and the participants were instructed to successfully complete the 360° and 540° turning tasks at the maximum speed three times.

The orientation of body segments in a global coordinate system was captured using nine cameras (Vicon MX-T10, Oxford Metrics, Oxford, UK). The data sampling frequency was 100 Hz [24]. Three-dimensional motion data were captured using the Vicon Nexus software (version 1.83; Oxford Metrics, UK). The appropriate metrics were measured bilaterally to estimate the joint kinematic data. The plug-in gait marker set was attached with 39 14-mm spherical reflective markers, which is a modified version of the Helen Hayes marker set [25].

**Figure 2:**
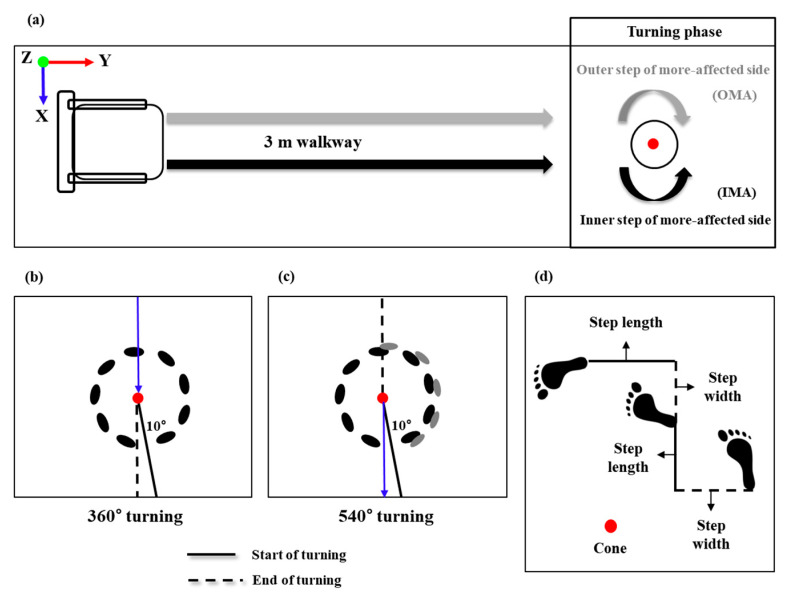
Schematic of the data collection and analysis phase. (**a**) The 360° turning task involved sitting on the chair, getting up from the chair, turning around the cone at the 3-m point, and reaching the final target point as quickly as possible. The 540° turning task involved sitting on the chair, getting up from the chair, turning around the cone at the 3-m point, and reaching the chair as quickly as possible. (**b**,**c**) In the analysis phase of the 360° and 540° turning tasks, the start event was defined as the event when the angle between the pelvic vector and the left and right vectors passed 10°. After the turning movement was completed, the event when the two vectors completed 360° and 540° was defined as the end of the rotation. (**d**) Step width was defined as the length between the initial foot heel contact of one leg and the initial foot heel contact of the other leg. The inner and outer step lengths were defined as the lengths of the initial foot heel contact of the left/right leg and the initial foot heel contact of the other leg, respectively.

The 360° and 540° turning tasks were conducted with maximum speed, which was defined as the fastest speed at which the patients choose to perform the tasks, in both the directions of the inner step of the more-affected side (IMA) and the outer step of the more-affected side (OMA). The turning directions for the more-affected sides (IMA and OMA) were classified by a neurologist. The 360° and 540° turning tasks with maximum speed used in this study were modified from the TUG test (Figure 2).

### 2.3. Data Analysis

The 360° and 540° turning data was a fourth-order Butterworth low-pass filtered with a cutoff frequency of 10 Hz [24]. The measurements were performed three times and the averaged value was used for the analysis.

The variables for the analysis were the total steps, total duration, step width, inner and outer step lengths, area of the 95% confidence interval (CI), and the anterior–posterior (AP) and medial–lateral (ML) root mean square (RMS) distances of the center of mass (COM) during the 360° and 540° turning tasks performed at maximum speed. A weighted sum of the COM of all segments was calculated, where the segments were defined by markers. The COM was the COM of all modeled segments. In addition, the area of the 95% CI (Equation (1)) and the AP and ML RMS distances of the COM on the horizontal plane (Equation (2)) were computed as [26]:

(1) Area=πab,

where a and b denote the major and minor axis, respectively.

(2) $$a={\left[3\left({\mathrm{SD}}_{\mathrm{AP}}^{2}+{\;\mathrm{SD}}_{\mathrm{ML}}^{2}+D\right)\right]}^{\frac{1}{2}};\;b={\left[3\left({\mathrm{SD}}_{\mathrm{AP}}^{2}+{\;\mathrm{SD}}_{\mathrm{ML}}^{2}-D\right)\right]}^{\frac{1}{2}},$$

where ${\mathrm{SD}}_{\mathrm{AP}}$ and ${\mathrm{SD}}_{\mathrm{ML}}$ denote the standard deviations of the AP and ML RMS distances of the COM, respectively.

(3) $$D=[\left({\mathrm{SD}}_{\mathrm{AP}}^{2}+{\;\mathrm{SD}}_{\mathrm{ML}}^{2}\right)-4{\left({\mathrm{SD}}_{\mathrm{AP}}^{2}{\mathrm{SD}}_{\mathrm{ML}}^{2}-{\mathrm{SD}}_{\mathrm{APML}}^{2}\right]}^{\frac{1}{2}},$$

where D denotes the distance from the center to the focus for the AP and ML RMS distances of the COM.

(4) $${\mathrm{SD}}_{\mathrm{APML}}^{\;}=\frac{1}{N}{\left(\sum_{n=1}^{N}X_{\mathrm{AP}\left(n\right)}X_{\mathrm{ML}\left(n\right)}\right)}^{\;},$$

where $X_{\mathrm{AP}}$ and $X_{\mathrm{ML}}$ denote the position of the AP and ML of the COM, respectively.

(5) $${\mathrm{RMS}}_{\mathrm{AP}}={\left[\frac{1}{N}\sum_{n=1}^{N}{\left(X_{\mathrm{AP}\left(n\right)}-{\bar{X}}_{\mathrm{AP}}\right)}^{2}\right]}^{\frac{1}{2}};\;{\mathrm{RMS}}_{ML}={\left[\frac{1}{N}\sum_{n=1}^{N}{\left(X_{\mathrm{ML}\left(n\right)}-{\bar{X}}_{\mathrm{ML}}\right)}^{2}\right]}^{\frac{1}{2}},$$

where ${\bar{X}}_{\mathrm{AP}}$ and ${\bar{X}}_{\mathrm{ML}}$ denote the mean of AP and ML of the COM, respectively.

### 2.4. Statistical Analysis

The Shapiro–Wilk test revealed that data followed a normal distribution. A two-way analysis of variance (ANOVA) with repeated measures was performed to analyze the differences between the groups (PD patients compared with controls and freezers compared with non-freezers) and between the turning directions (IMA and OMA) during the 360° and 540° turning tasks at maximum speed. *Post-hoc* tests were performed using the independent sample t-test between the groups and the paired samples t-test was used to analyze the turning directions. To compare the differences of physical characteristics between all participants, one-way ANOVAs were performed.

In addition, the responsiveness between the groups and the turning directions were expressed as the effect size (ES). Univariate and multivariate logistic regression analyses and stepwise binary logistic regression analysis were performed to identify the classifier variables for PD patients and freezers, and for the turning directions. Classifier variables were expressed as odds ratios (OR) with a 95% CI (min to max). All statistical analyses were performed using SPSS 22.0 (SPSS Inc., Chicago, IL, USA), and the statistical significance was set at 0.05.

## 3. Results

### 3.1. Differences between PD Patients and Controls

In the results of the 360° turning task at the maximum speed, the total steps (IMA, *p* = 0.035), total duration (IMA, *p* = 0.006; OMA, *p* = 0.047), inner (IMA, *p* = 0.003; OMA, *p* < 0.001) and outer (IMA, *p* = 0.001; OMA, *p* < 0.001) step lengths, and ML RMS distance of the COM (IMA, *p* = 0.042; OMA, *p* = 0.016) of the turning tasks in the directions of the IMA and OMA showed significant differences between PD patients and controls (Table 2). In the results of the 540° turning task at the maximum speed, the inner (OMA, *p* = 0.018) and outer (IMA, *p* = 0.001) step lengths and AP RMS distance of the COM (IMA, *p* = 0.042; OMA, *p* = 0.016) of the turning tasks in the directions of the IMA and OMA showed significant differences between the PD patients and controls (Table 3).

### 3.2. Classifier Variables for PD Patients and Controls

Stepwise binary logistic regression analysis for the PD patients and controls revealed that the total duration (OR: 0.271; 95% CI: 0.077–0.957; R_N_^2^ = 0.532; *p* = 0.043) and outer step length (OR: 4.826; 95% CI: 1.248–18.659; R_N_^2^ = 0.532; *p* = 0.023) of the turning task performed in the direction of the IMA and inner step length (OR: 7.218; 95% CI: 1.831–28.463; R_N_^2^ = 0.607; *p* = 0.005) of the turning task performed in the direction of the OMA were significantly different during the 360° turning task at the maximum speed. In addition, the outer step length (OR: 5.642; 95% CI: 1.578–20.167; R_N_^2^ = 0.390; *p* = 0.008) of the turning task performed in the direction of the IMA and inner step length (OR: 2.984; 95% CI: 1.092–8.152; R_N_^2^ = 0.223; *p* = 0.033) of the turning task performed in the direction of the OMA were significantly different during the 540° turning task at the maximum speed.

### 3.3. Differences between Freezers and Non-Freezers

**Figure 3:**
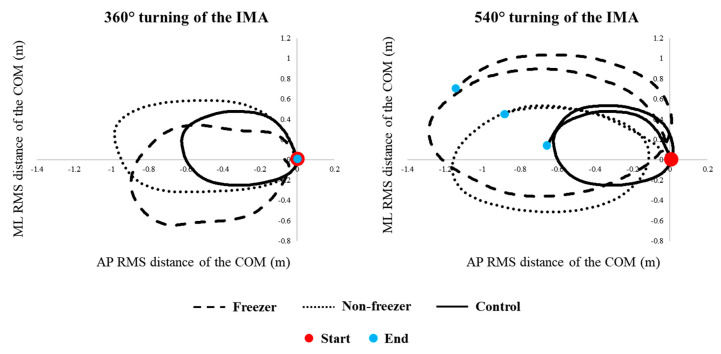
Single participant trial of freezers, non-freezers, and controls during the 360° and 540° turning tasks. This figure presented the horizontal trajectory of the center of mass presented by large (a freezer) and small (a non-freezer) solid dots, and full line (a control), respectively.

The AP (360° turn, *p* < 0.001; 540° turn, *p* < 0.001) and ML (360° turn, *p* < 0.001; 540° turn, *p* < 0.001) RMS distances of the COM in the turning task performed in the direction of the IMA (Figure 3) and the ML RMS distance of the COM (360° turn, *p* = 0.003; 540° turn, *p* = 0.013) in the turning task performed in the direction of the OMA were significantly different for freezers and non-freezers during the 360° and 540° turning tasks at the maximum speed (Table 4 and Table 5).

### 3.4. Classifier Variables for Freezers and Non-Freezers

Stepwise binary logistic regression analysis for freezers and non-freezers showed that the ML RMS distance of the COM in the turning tasks performed in the directions of the IMA (OR: 10.188; 95% CI: 1.699–61.100; R_N_^2^ = 0.596; *p* = 0.011) and OMA (OR: 0.193; 95% CI: 0.053–0.708; R_N_^2^ = 0.437; *p* = 0.013) were significantly different during the 360° turning task at the maximum speed. In addition, the inner step length (OR: 0.047; 95% CI: 0.004–0.560; R_N_^2^ = 0.665; *p* = 0.016) of the turning task performed in the direction of the IMA and the ML RMS distance of the COM (OR: 0.276; 95% CI: 0.086–0.883; R_N_^2^ = 0.324; *p* = 0.030) in the turning task performed in the direction of the OMA were significantly different during the 540° turning task at the maximum speed.

### 3.5. Differences between Turning Characteristics in the Directions of the IMA and OMA

In the results of the PD patients, the step width (*p* = 0.042) and AP (*p* = 0.009) and ML (*p* = 0.002) RMS distances of the COM in the turning tasks performed in the direction of the IMA were significantly higher than those in the direction of the OMA during the 360° turning task at the maximum speed (Table 2). In addition, the total steps (*p* = 0.008) and AP (*p* = 0.005) and ML (*p* = 0.003) RMS distances of the COM in the turning tasks performed in the direction of the IMA were significantly higher than those in the direction of the OMA during the 540° turning task at the maximum speed (Table 3). In the results of the freezers, the total steps (*p* = 0.026) and inner step length (*p* = 0.007) of the turning tasks performed in the direction of the IMA were significantly higher than those in the direction of the OMA during the 540° turning task at the maximum speed (Table 5).

### 3.6. Classifier Variables for Turning Tasks in the Directions of the IMA and OMA

Stepwise binary logistic regression analysis for the PD patients revealed that the ML RMS distance of the COM was significantly different in the turning task performed in the direction of the IMA during the 360° (OR: 0.000; 95% CI: 0.000–0.005; R_N_^2^ = 0.301; *p* = 0.002) and 540° (OR: 0.000; 95% CI: 0.000–0.021; R_N_^2^ = 0.231; *p* = 0.004) turning tasks at the maximum speed. In addition, the results for freezers revealed that the ML RMS distance of the COM was significantly different in the turning direction of the IMA during the 360° (OR: 0.000; 95% CI: 0.000–0.010; R_N_^2^ = 0.405; *p* = 0.005) and 540° (OR: 0.000; 95% CI: 0.000–0.014; R_N_^2^ = 0.426; *p* = 0.004) turning tasks at the maximum speed. Significant differences were found for the total steps (OR: 0.755; 95% CI: 0.574–0.993; R_N_^2^ = 0.426; *p* = 0.045) in the 540° turning task performed in the direction of the IMA at the maximum speed.

## 4. Discussion

### 4.1. Differences between PD Patients and Controls, and Freezers and Non-Freezers

We found that PD patients showed a significantly longer total duration, shorter inner and outer step lengths, and greater ML RMS distance of the COM during the 360° turning task at the maximum speed compared to those of the controls. The total duration and outer step length of the 360° and 540° turning tasks performed in the direction of the IMA and the inner step length of the 360° and 540° turning tasks performed in the direction of the OMA at the maximum speed were revealed as the classifier variables for the PD patients. In addition, the freezers showed significantly greater AP and ML RMS distances of the COM in the 360° and 540° turning tasks performed in the direction of the IMA, and greater ML RMS distance of the COM in the 360° and 540° turning tasks performed in the direction of the OMA at the maximum speed in comparison with those of the non-freezers. The ML RMS distance of the COM in the 360° and 540° turning tasks performed in the directions of the IMA and OMA at the maximum speed were revealed as the classifier variables for the freezers. These results demonstrated our hypothesis that turning tasks under challenging conditions might be more difficult for PD patients than for controls, and for freezers than for non-freezers.

Turning tasks threaten the stability of PD patients more than any other freezing trigger, and they require the precise control of each limb [26]. Therefore, PD patients may experience greater challenge and distraction in performing turning tasks. Previous studies have reported that PD patients showed significantly greater total steps, wider step width, shorter step length, and slower turning speed to maintain their center of gravity between the two feet [27]. Increased turning angle and speed were observed during 90° and 180° turning tasks at the preferred speed [28], 180° turning tasks at comfortable and faster speeds [29], and 30° to 180° turning tasks at the self-selected preferred, slower, and faster speeds [15]. Thus, these turning characteristics may be a compensatory strategy for postural instability in PD patients [30].

The motor cortex closely cooperates with the basal ganglia, and the cerebellum is involved in some functions such as attention, executive and visuospatial functions, and control motor tasks [31]. The motor symptoms in PD patients are the results of incrementally impaired motor functions and impaired basal ganglia cells in the substantia nigra, which initiate and control body movements and balance [32]. Further, the degeneration of the substantia nigra is reflected as a loss of dopaminergic innervation, which may influence the control of automatized behavior. Therefore, PD patients may experience difficulty in automatic movements without attention [33]. The abnormalities in the basal ganglia or spinal cord circuit behavior have been suggested to be important factors influencing PD patients because of the greater decline in voluntary contractions [34]. The turning tasks thus require more attention and involve greater interlimb coordination, increased coupling between posture and gait [35], and modifications of locomotor patterns requiring frontal lobe cognitive and executive functions that control postural transitions [36]. Thus, these results suggest that challenging turning tasks may indicate postural instabilities, which imply that PD patients are more likely to be vulnerable to functional impairments [37] because turning is inherently asymmetrical and cannot stem from central pattern generators unlike walking in a straight line [38].

The results of our study show that PD patients might experience greater difficulty in 360° and 540° turning tasks in the directions of the IMA and OMA at the maximum speed in comparison with the controls, as evidenced by longer total turn durations, shorter inner and outer step lengths, and greater ML RMS distances of the COM (Figure 3). These differences were associated with relatively small effect sizes (d = 0.01–0.32). In addition, this study demonstrated classifier variables for the PD patients. We found that the total duration and the inner and outer step length may be considered when comparing PD patients and controls in addition to classifier variables to identify turning difficulty during the 360° and 540° turning tasks performed in the direction of the IMA and OMA at the maximum speed. Our results are useful for determining the decline in dynamic stability and the posture control of PD patients, and they can potentially be used in clinical environments to measure the effectiveness of interventions meant to prevent risk of falls.

In previous studies, the freezers showed higher total steps, longer total durations, slower turning speeds, wider step widths, and shorter step lengths than the non-freezers, as well as increased turning angles and speeds while performing tasks such as 0°, 90°, 120°, 180°, and 360° turning tasks at the preferred and faster speeds [1,6,16]. Turning difficulty has been related to disorders of the frontocortical areas, which may be more severe in freezers than in non-freezers because freezers present a lower supplementary motor area activity than non-freezers [39]. The dysfunction of the supplementary motor area may require the functional reorganization of the spinal circuitry, given the abnormal projections from this area through the reticulospinal tract [40]. As the reticulospinal tract connects to the spinal interneurons that mediate one of the most powerful spinal inhibitory mechanisms, presynaptic inhibition [41], it is possible that presynaptic inhibition during challenging turning tasks may be deficient in freezers compared to those in controls and non-freezers. Therefore, increasing the turning angle and turning speed might pose additional difficulties in threatening situations, where dynamic stability while performing turning tasks with a small turning angle at the normal speed is potentially related to difficulties in coupling the inter-limb coordination and postural control [1,6]. In addition, such turning tasks in freezers may lead to an increased risk of fall because these conditions are not commonly experienced in daily life and therefore require greater attention and postural stability [15,29,42]. 

Further, our results show that freezers might experience greater difficulty in turning at 360° and 540° angles in the directions of the IMA and OMA at the maximum speed compared to non-freezers, as evidenced by the greater AP and ML RMS distances of the COM (Figure 3). These differences were associated with a relatively moderate effect size (d = 0.27–0.54). In addition, we found that the ML RMS distance of the COM may be considered when comparing freezers and non-freezers in addition to classifier variables to identify disease severity during the 360° and 540° turning tasks performed in the direction of the IMA and OMA at the maximum speed. Therefore, we suggest that preventive training to reduce risk of falling may require practicing around a larger radius to complete a turn [28,43].

### 4.2. Differences between Turning Characteristics in the Directions of the IMA and OMA

We found that PD patients showed significantly greater step widths, higher total steps, and greater AP and ML RMS distances of the COM in the 360° and 540° turning tasks performed at the maximum speed in the direction of the IMA than those in the direction of the OMA. In addition, the ML RMS distance of the COM while turning by 360° and 540° at the maximum speed in the direction of the IMA was revealed as the classifier variable for the PD patients and controls. The 540° turning task performed by the freezers at the maximum speed revealed significantly higher total steps and shorter inner step length in the direction of the IMA than those in the direction of the OMA. Moreover, the ML RMS distance of the COM during the 360° turning task in the direction of the IMA and the total steps and ML RMS distance of the COM during the 540° turning task in the direction of the IMA at the maximum speed were revealed as the classifier variables for freezers and non-freezers. The study results proved our hypothesis that turning in the direction of the more-affected side might present more difficulties in PD patients and freezers.

Only one study on turning tasks toward the more-affected and less-affected sides in PD patients and freezers [14] reported that such patients showed higher turning steps and a greater duration of turn while turning 180° and 360° toward the more-affected side. Thus, PD patients and freezers might experience greater difficulty in turning toward the more-affected side [14], which increases the risk of falls [9].

Previous studies reported that the less-affected side assumes the function of the more-affected side during a turning task, as a compensatory strategy between the lower limbs in PD patients and freezers [9,44]. This asymmetry in performing the turning tasks is responsible for the motor manifestation characteristics of PD patients [45]. The cause of this asymmetry is not known [46]. However, it might reflect a different vulnerability of the substantia nigra pars compacta neurons to the factors involved in the generation of the disorder. Therefore, these results suggest that freezers with higher asymmetry may experience greater difficulty than non-freezers and controls with symmetrical motor involvement [45]. The motor manifestations are predominantly observed while performing challenging turning tasks [46].

Lee et al. [47] suggested that the more-affected side of the PD patients tends to be affected predominantly throughout disease progression. Therefore, this dominant tendency may promote greater motor deficits on the more-affected side. These results suggest that turning difficulty may be related to asymmetry between the more-affected and less-affected sides of the lower limbs [47]. Moreover, shorter step lengths may be related to weakened muscle strength, which could influence the alterations in the lower limb kinematics [48]. In addition, freezers may have greater difficulty in adapting postural control during turning and switching their motor pattern to meet the asymmetrical demands while turning in the direction of the more-affected side [5]. These results suggest that the efforts made by PD patients and freezers to increase the postural stability during the turning tasks may help compensate the reduced lower limb strength of the more-affected side [48].

The findings presented in this study have some important implications. First, PD patients and freezers might experience greater difficulty in turning toward the direction of the more-affected side, which may be useful in the clinical assessment of the disease severity and FOG. Thus, fall prevention training may require focus on improving the turning performances, particularly turning toward the more-affected side, by practicing wider turns, turning more slowly, or practicing to sustain a wider base of support. Second, the ML RMS distance of the COM was shown as a marker that can distinguish between the severity of the motor symptoms in PD patients and controls, or freezers and non-freezers.

However, our study presents some limitations. First, the sample size was small for the generalization of the results obtained. Although a different sample size was used in this study, the lack of influences may have been obtained in the results. Second, the comparison of antiparkinsonian medication effects was not performed while evaluating the turning tasks of this study. Third, this study was based on difficult turning tasks that do not appear in daily life activities. Finally, this study, which used 39 markers, was considered to observe turning movements such as upper and lower limb coordination and posture control and dynamic stability of PD patients. However, we did not consider these results of upper limbs movement in our study because we focused on only lower limb movements.

## 5. Conclusions

Our study investigated the characteristics of turning performance in the direction of the more-affected side in PD patients in comparison with controls, and in freezers in comparison with non-freezers during 360° and 540° turning tasks at the maximum speed. We found that the PD patients and freezers exhibited higher turning difficulty during challenging turning tasks such as those requiring increasing turning angles and speeds. Further, PD patients and freezers may be in greater danger of falling while turning in the direction of the more-affected side. Therefore, turning tasks involving 360° and 540° turning tasks in the direction of the IMA and OMA at the maximum speed may be useful for evaluating the turning characteristics to distinguish between PD patients and freezers. Further studies are needed to evaluate turning characteristics according to motor symptoms in PD patients at more various conditions during turning tasks.

## Figures and Tables

**Table 1:** Physical characteristics of all participants.

	PD Patients		Controls (n = 12)	*p*-Value
	Freezers (n = 12)	Non-Freezers (n = 12)		
Sex (male/female)	**7/5**	8/4	6/6	-
Age (years)	66.67 ± 4.38	68.83 ± 6.00	68.25 ± 3.47	0.517
Height (cm)	158.83 ± 9.08	157.73 ± 7.22	160.30 ± 9.29	0.765
Body weight (kg)	57.88 ± 8.97	61.07 ± 8.43	61.53 ± 9.54	0.563
BMI (kg/m^2^)	22.86 ± 2.24	24.55 ± 3.13	23.84 ± 2.20	0.281
MMSE (scores)	27.33 ± 2.06	26.67 ± 2.57	26.00 ± 1.76	0.330
Disease duration (years)	9.83 ± 4.26	5.96 ± 1.83	-	**0.008**
Treatment duration (years)	8.95 ± 4.35	3.52 ± 2.26	-	**0.001**
UPDRS Total (scores)	60.47 ± 9.59	38.13 ± 5.90	-	**<0.001**
UPDRS III (scores)	33.38 ± 6.16	27.96 ± 4.38	-	**0.023**
Hoehn and Yahr scale	2.55 ± 0.27	2.38 ± 0.31	-	0.176
NFOGQ (scores)	19.18 ± 5.62	-	-	-
L-Dopa equivalent dose (mg/day)	1142.50 ± 418.20	682.92 ± 239.17	-	**0.003**
More affected side (left/right)	11/1	8/4	All right-handed	-

All data represent the mean ± standard deviation; PD: Parkinson’s disease; BMI: Body mass index; MMSE: Mini- mental state examination; UPDRS: Unified Parkinson’s disease rating scale; L-dopa: Levodopa. Boldface denotes a significant difference between freezers and non-freezers. Significant difference: *p* < 0.05.

**Table 2:** Comparison of turning characteristics of PD patients and controls for the 360° turning task at maximum speed.

		PD	Controls	*p^a^*-Value	ES
Total steps	**IMA**	9.17 ± 2.59	7.39 ± 1.52	**0.035**	0.09
	**OMA**	9.96 ± 2.53	9.11 ± 2.14	0.328	0.24
	*p^b^*-value	0.060	0.037		0.04
Total duration (s)	**IMA**	4.34 ± 1.18	3.25 ± 0.66	**0.006**	0.18
	**OMA**	4.67 ± 2.03	3.42 ± 0.70	**0.047**	0.03
	*p^b^*-value	0.338	0.566		0.00
Step width (m)	**IMA**	0.13 ± 0.08	0.17 ± 0.08	0.133	0.18
	**OMA**	0.09 ± 0.04	0.15 ± 0.07	**0.006**	0.11
	*p^b^*-value	**0.042**	0.353		0.00
Inner step length (m)	**IMA**	0.40 ± 0.07	0.47 ± 0.05	**0.003**	0.32
	**OMA**	0.41 ± 0.06	0.49 ± 0.05	**<0.001**	0.05
	*p^b^*-value	0.605	0.156		0.01
Outer step length (m)	**IMA**	0.40 ± 0.07	0.49 ± 0.06	**0.001**	0.32
	**OMA**	0.40 ± 0.07	0.49 ± 0.04	**<0.001**	0.01
	*p^b^*-value	0.588	0.914		0.00
Area of horizontal plane of the COM (m^2^)	**IMA**	2.43 ± 0.59	2.67 ± 0.35	0.140	0.14
	**OMA**	2.23 ± 0.69	2.56 ± 0.31	0.050	0.03
	*p^b^*-value	0.347	0.149		0.00
Anterior-posterior RMS distance of the COM (m)	**IMA**	0.29 ± 0.13	0.24 ± 0.06	0.258	0.01
	**OMA**	0.20 ± 0.08	0.22 ± 0.04	0.403	0.14
	*p^b^*-value	**0.009**	0.218		0.07
Medial-lateral RMS distance of the COM (m)	**IMA**	0.29 ± 0.13	0.22 ± 0.05	**0.042**	0.01
	**OMA**	0.16 ± 0.06	0.20 ± 0.03	**0.016**	0.19
	*p^b^*-value	**0.002**	0.182		0.12

All data represent the mean ± standard deviation (95% confidence interval). *p^a^*-value (groups): Independent t-tests; *p^b^*-value (turning direction): Paired samples t-tests; ES: Effect size; IMA: Inner step of the more affected side; OMA: Outer step of the more affected side; COM: Center of mass; RMS: Root mean square. Boldface indicates a significant difference (*p* < 0.05).

**Table 3:** Comparison of turning characteristics of PD patients and controls for the 540° turning task at maximum speed.

		PD	Controls	*p^a^*-Value	ES
Total steps	**IMA**	12.33 ± 2.64	10.61 ± 2.12	0.058	0.03
	**OMA**	10.75 ± 2.55	10.89 ± 3.11	0.886	0.05
	*p^b^*-value	**0.008**	0.760		0.09
Total duration (s)	**IMA**	6.51 ± 1.77	6.30 ± 3.01	0.789	0.02
	**OMA**	6.30 ± 3.14	5.43 ± 1.55	0.372	0.02
	*p^b^*-value	0.759	0.381		0.01
Step width (m)	**IMA**	0.14 ± 0.08	0.16 ± 0.06	0.377	0.02
	**OMA**	0.12 ± 0.04	0.12 ± 0.07	0.945	0.08
	*p^b^*-value	0.390	0.158		0.01
Inner step length (m)	**IMA**	0.44 ± 0.09	0.50 ± 0.11	0.141	0.20
	**OMA**	0.41 ± 0.10	0.49 ± 0.07	**0.018**	0.01
	*p^b^*-value	0.351	0.915		0.01
Outer step length (m)	**IMA**	0.39 ± 0.08	0.48 ± 0.05	**0.001**	0.19
	**OMA**	0.42 ± 0.09	0.47 ± 0.05	0.100	0.02
	*p^b^*-value	0.057	0.180		0.10
Area of horizontal plane of the COM (m^2^)	**IMA**	2.40 ± 0.59	2.48 ± 0.42	0.645	0.06
	**OMA**	2.05 ± 0.57	2.33 ± 0.27	0.058	0.10
	*p^b^*-value	0.068	0.114		0.02
Anterior-posterior RMS distance of the COM (m)	**IMA**	0.28 ± 0.13	0.23 ± 0.09	0.315	0.01
	**OMA**	0.18 ± 0.06	0.19 ± 0.03	0.577	0.20
	*p^b^*-value	**0.005**	0.109		0.04
Medial-lateral RMS distance of the COM (m)	**IMA**	0.26 ± 0.13	0.19 ± 0.03	0.059	0.03
	**OMA**	0.15 ± 0.06	0.19 ± 0.03	0.058	0.14
	*p^b^*-value	**0.003**	0.660		0.13

All data represent the mean ± standard deviation (95% confidence interval). *p^a^*-value (groups): Independent t-tests; *p^b^*-value (turning direction): Paired samples t-tests. Boldface indicates a significant difference (*p* < 0.05).

**Table 4:** Comparison of turning characteristics of freezers and non-freezers for the 360° turning task at maximum speed.

		Freezers	Non-Freezers	*p^a^*-Value	ES
Total steps	**IMA**	9.78 ± 2.52	8.56 ± 2.61	0.256	0.02
	**OMA**	9.94 ± 2.76	9.97 ± 2.40	0.979	0.16
	*p^b^*-value	0.750	**0.034**		0.11
Total duration (s)	**IMA**	4.31 ± 1.09	4.37 ± 1.32	0.899	0.02
	**OMA**	5.07 ± 2.34	4.27 ± 1.67	0.346	0.04
	*p^b^*-value	0.211	0.787		0.07
Step width (m)	**IMA**	0.14 ± 0.10	0.12 ± 0.05	0.503	0.00
	**OMA**	0.09 ± 0.04	0.10 ± 0.03	0.424	0.18
	*p^b^*-value	0.058	0.407		0.05
Inner step length (m)	**IMA**	0.39 ± 0.08	0.41 ± 0.06	0.554	0.05
	**OMA**	0.39 ± 0.07	0.42 ± 0.05	0.212	0.01
	*p^b^*-value	0.935	0.226		0.02
Outer step length (m)	**IMA**	0.39 ± 0.06	0.41 ± 0.09	0.572	0.01
	**OMA**	0.40 ± 0.06	0.41 ± 0.08	0.837	0.01
	*p^b^*-value	0.327	0.957		0.02
Area of horizontal plane of the COM (m^2^)	**IMA**	2.39 ± 0.44	2.47 ± 0.72	0.722	0.02
	**OMA**	2.38 ± 0.43	2.07 ± 0.86	0.276	0.04
	*p^b^*-value	0.969	0.349		0.04
Anterior-posterior RMS distance of the COM (m)	**IMA**	0.20 ± 0.05	0.17 ± 0.03	**<0.001**	0.28
	**OMA**	0.20 ± 0.05	0.20 ± 0.11	0.887	0.38
	*p^b^*-value	0.674	**0.002**		0.41
Medial-lateral RMS distance of the COM (m)	**IMA**	0.19 ± 0.05	0.17 ± 0.03	**<0.001**	0.27
	**OMA**	0.19 ± 0.04	0.12 ± 0.06	**0.003**	0.54
	*p^b^*-value	0.818	**<0.001**		0.53

All data represent the mean ± standard deviation (95% confidence interval). *p^a^*-value (groups): Independent t-tests; *p^b^*-value (turning direction): Paired samples t-tests. Boldface indicates a significant difference (*p* < 0.05).

**Table 5:** Comparison of turning characteristics of freezers and non-freezers for the 540° turning task at maximum speed.

		Freezers	Non-Freezers	*p^a^*-Value	ES
Total steps	**IMA**	12.72 ± 2.25	11.94 ± 3.03	0.482	0.00
	**OMA**	10.61 ± 2.55	10.89 ± 2.66	0.797	0.28
	*p^b^*-value	**0.026**	0.168		0.04
Total duration (s)	**IMA**	5.89 ± 1.00	6.29 ± 1.02	0.340	0.05
	**OMA**	6.23 ± 3.52	6.37 ± 2.86	0.912	0.00
	*p^b^*-value	0.739	0.917		0.05
Step width (m)	**IMA**	0.15 ± 0.11	0.13 ± 0.05	0.456	0.00
	**OMA**	0.11 ± 0.04	0.14 ± 0.03	**0.043**	0.04
	*p^b^*-value	0.210	0.526		0.09
Inner step length (m)	**IMA**	0.51 ± 0.06	0.38 ± 0.07	**<0.001**	0.06
	**OMA**	0.38 ± 0.10	0.45 ± 0.07	0.055	0.07
	*p^b^*-value	**0.007**	**0.008**		0.48
Outer step length (m)	**IMA**	0.38 ± 0.07	0.40 ± 0.09	0.628	0.06
	**OMA**	0.39 ± 0.07	0.45 ± 0.10	0.106	0.16
	*p^b^*-value	0.555	0.072		0.10
Area of horizontal plane of the COM (m^2^)	**IMA**	2.22 ± 0.48	2.59 ± 0.65	0.127	0.00
	**OMA**	2.21 ± 0.56	1.90 ± 0.56	0.182	0.16
	*p^b^*-value	0.949	0.051		0.15
Anterior-posterior RMS distance of the COM (m)	**IMA**	0.19 ± 0.05	0.16 ± 0.02	**<0.001**	0.36
	**OMA**	0.19 ± 0.06	0.17 ± 0.07	0.646	0.42
	*p^b^*-value	0.925	0.550		0.41
Medial-lateral RMS distance of the COM (m)	**IMA**	0.18 ± 0.05	0.15 ± 0.03	**<0.001**	0.27
	**OMA**	0.18 ± 0.06	0.12 ± 0.05	**0.013**	0.49
	*p^b^*-value	0.810	**0.001**		0.50

All data represent the mean ± standard deviation (95% confidence interval). *p^a^*-value (groups): Independent t-tests; *p^b^*-value (turning direction): Paired samples t-tests. Boldface indicates a significant difference (*p* < 0.05).

## Data Availability

The datasets generated and/or analyzed during the current study are not publicly available due to intellectual property reasons, but these are available upon a reasonable request.

## Supplementary Materials

The following are available online at https://www.mdpi.com/1424-8220/20/11/3098/s1, Supplementary File S1: IRB number, Supplementary File S2: Research data (raw data), Supplementary Table S1: List of levodopa-equivalent doses.
