# Supplementary material for: Turning Characteristics of the More-Affected Side in Parkinson’s Disease Patients with Freezing of Gait
Source: Sensors (Basel). 2020 May 30;20(11):3098. doi: 10.3390/s20113098 (PMC7309092; doi:10.3390/s20113098)
Supplement: Supplementary file 1 [file sensors-20-03098-s001.zip › Supplementary file S1. IRB number.pdf]

심의결과 통지서

우 49201 부산광역시 서구 대신공원로 26 ☎051-240-2572,2577 전송 : 051-231-2140

수신	의뢰기관	없음			
	연구책임자	소속	신경과	성명·직위	천상명 · 부교수

과제번호(IRB No.)	DAUHIRB-17-033			
연구 과제명	국문	보행동결이 있는 파킨슨병 환자와 비전형적 파킨슨 증후군 환자의 회전 보행 특성 분석과 낙상 예측 요인 개발		
	영문	Analysis of Turning Characteristics for Parkinson's Disease People with Freezing of Gait and Atypical parkinsonism and Development of Factors for Fall Prediction		
연구예정기간	IRB 승인일로부터 ~ 2017년 08월 31일		연구승인유효기간	2017년 08월 31일
심의 구분	●신속심의 ○정규심의		심의 일자	2017년 02월 07일
지속심의주기	○3개월 ○6개월 ○1년 ●기타			
심의결과	●승인 ○시정승인 ○보완 후 신속심의 ○보완 후 정기심의<br>○반려 ○승인된 임상시험의 중지 또는 보류			
심의 의견				
시정승인 내용을 검토 확인후 승인합니다.				
제출서류 목록				
연구계획서요약<br>연구계획서<br>Version : Version 2.0 2017.01.04 (연구계획서 Version 2.0.pdf)<br>연구계획서 체크리스트<br>연구대상자 설명문 및 동의서<br>Version : Version 2.0 2017.01.04 (연구대상자용 설명문 및 동의서 Version 2.0.pdf)<br>연구자용 연구대상자 동의서 체크리스트<br>증례기록서<br>Version : Version 2.0 2017.01.04 (CRF-신체조성_측정지_Version_2.0.pdf)<br>연구대상자 모집광고 문안<br>Version : Version 2.0 2017.01.04 (외래환자 모집 자료 Version 2.0.pdf)<br>Delegation Log<br>Version : Version 2.0 2017.01.04 (연구진 Delegation Log Version 2.0.pdf)<br>연구책임자 이력서 전자서식				

* 본 임상연구심의위원회는 국제표준화추진위원회(ICH), 약사법, 의료기기법, 생명윤리 및 안전에 관한 법률 등 관련 법류를 준수합니다.

* 본 연구과 이해상충관계가 있는 위원이 있을 경우 해당 위원은 연구의 심의에서 배제하였습니다.

* 심의결과가 시정승인, 보완(신속심의,보완(정기심의)인 경우 보완심의신청서를 제출하시면 신속 또는 정기심의를 거쳐 최종승인을 받을 수 있습니다.

* 심의결과에 이의가 있을 경우 이의신청서를 작성하여 제출하여 주시기 바랍니다.

2017년 02월 07일

동아대학교병원 임상연구심의위원회 위원장

연구자의 책임과 의무
1. 국제표준화 추진회의(ICH), 의약사법, 의료기기법, 생명윤리 및 안전에 관한 법률 등 관련 법규를 준수하여야 합니다.<br>2. 계획서의 승인 이전에 연구 대상자를 해당 임상연구에 참여시키는 것을 금지합니다.<br>3. 연구 대상자에게 발생한 즉각적 위험 요소의 제거가 필요한 경우를 제외하고는 변경계획서에 대한 승인이 내려지기 이전에 원 계획서와 다르게 임상시험을 실시하는 것을 금지합니다.<br>4. 계획서변동/위반이 연구대상자의 안전과 복지나 연구 자료의 아전성에 악영향을 미칠 수 있을 때는 연구자는 이를 인지한지 7일 이내에 위원회에 보고하여야 합니다.<br>**-변동**(Deviation)은 승인된 연구계획과는 다르게 진행되었지만 환자의 권리, 안전, 복지나 연구의 완전성에 직접적인 영향을 끼치지 않는 경우를 말합니다.<br>**-위반**(Violation)은 승인된 연구계획과는 다르게 진행되어 환자의 권리, 안전, 복지, 연구 및 결과 자료에 직접적인 영향을 끼칠 수 있는 경우를 말합니다.<br>5. 위원회의 승인을 받은 연구 대상자 동의서를 사용하여야 합니다.<br>: 연구대상자 설명문 및 동의서가 변경된 경우 이미 진행된 연구대상자에게도 변경된 연구대상자 설명문 및 동의서를 받으시기 바랍니다.<br>6. 모국어가 한국어가 아닌 연구 대상자들에게는 승인된 동의서를 연구 대상자의 모국어로 인증된 번역본을 사용할 것이며, 이러한 동의서 번역본은 반드시 위원회 승인을 받아야 합니다.<br>7. 연구진행에 있어 연구 대상자를 보호하기 위해 불가피한 경우를 제외하고 연구의 어떠한 변경이든 위원회의 사전 승인을 받고 수행할 것 연구 대상자들의 보호를 위해 취해진 어떠한 응급상황에서의 변경도 즉각 위원회에 보고하여야 합니다.<br>8. 연구 대상자에게 중대한 이상반응/의료기기 반응이 발생하였을 경우에는 위원회에 서면으로 보고하여야 합니다.<br>9. 임상시험 또는 연구 대상자의 안전에 대해 유해한 영향을 미칠 수 있는 어떠한 새로운 정보도 즉각적으로 위원회에 보고하여야 합니다.<br>10. 위원회의 요구가 있을 때에는 연구의 진행과 관련된 보고를 위원회에 제출하여야 합니다.<br>11. 연구 대상자 모집광고는 사용 전에 위원회로부터 승인을 획득하여야 합니다.<br>12. 강제 혹은 부당한 영향이 없는 상태에서 충분한 설명에 근거한 동의 과정을 수행할 것이며, 잠재적인 연구 대상자에 게 연구에의 참여여부를 고려할 수 있도록 충분히 기회를 제공하여야 합니다.<br>13. **위원회의 승인은 1년을 초과할 수 없습니다.** 따라서 위원회에서 요구한 지속심의 주기에 따라 지속심의를 신청하여야 합니다.<br>14. 연구가 종료된 경우 종료보고서를 제출하여야 합니다.<br>15. 연구를 조기종료 또는 일시중지 시켰을 경우 위원회에 즉시 일시중지보고서를 제출하여야 합니다.<br>16. 의뢰자는 시험기관의 장과 문서로서 임상시험계약을 체결하여야 합니다.<br>17. 임상시험 실시기관명은 해당 실시기관의 사전 서면동의 없이는 광고 등의 목적에 사용할 수 없습니다.<br>**18. 헬싱키 선언(제19조)에 따라 모든 임상시험은 첫 연구대상자 모집하기 전 공개적으로 접근이 가능한 임상연구등록시스템에 등록하여 이를 공개하여야 하며, 예를 들어, 질병관리본부에서 운영하는 임상연구정보서비스(CRIS, http:// cris.cdc.go.kr)를 이용하실 수 있습니다.**
